# Patient Safety Culture in the Context of Critical Care: An Observational Study

**DOI:** 10.3390/nursrep14030133

**Published:** 2024-07-19

**Authors:** Inês Oliveira, Cristina Costeira, Joana Pereira Sousa, Cátia Santos

**Affiliations:** 1 Médio Tejo Local Health Unit, 2304-909 Tomar, Portugal; 2 School of Health Science Campus 2, Polytechnic of Leiria, Morro do Lena, Alto do Vieiro, Apartado 4137, 2411-901 Leiria, Portugal; cristina.costeira@ipleiria.pt (C.C.); joana.sousa@ipleiria.pt (J.P.S.); 3 ciTechCare, Hub de Inovação, Rua das Olhalvas, Campus 5, Polytechnic of Leiria, 2414-016 Leiria, Portugal; 4 Health Sciences Research Unit: Nursing (UICISA: E), Nursing School of Coimbra (ESEnfC), 3004-011 Coimbra, Portugal

**Keywords:** patient safety, incident reporting, quality of healthcare, critical care, nursing

## Abstract

Background: A robust safety culture is essential for ensuring high-quality healthcare delivery. From a nursing perspective, especially among critical patients, it fosters ongoing improvement by highlighting areas that need attention. Aims: This study aimed to evaluate the perception of patient safety culture among nurses within the critical care environment. Methodology: An observational study was conducted at a central hospital in Portugal employing the Hospital Survey on Patient Safety Culture (HSPSC) questionnaire. Results: The study encompassed 57, nurses predominantly female (73.7%), aged 25–64. Most participants were general nurses (77.2%), with a significant proportion (61.4%) working in the emergency department and possessing an average tenure of 13 years at the facility. The perception of critical patient safety culture (CPSC) was predominantly positive (40.6%), varying by department, with intensive care nurses reporting the highest positivity rates. Teamwork was identified as a strong point, receiving 80.7% positivity, highlighting it as a well-established domain in the CPSC, whereas other domains were recognised as requiring enhancements. Conclusions: The study pinpointed both strengths and weaknesses within the CPSC, offering a foundation for developing targeted strategies to bolster patient safety culture in critical care settings.

## 1. Introduction

As an integral part of health policies, patient safety is a primary goal for all organisations, invariably associated with the quality of healthcare systems [1,2]. The right to health protection is a fundamental social right, protected under the terms of Article 64 of the Constitution of the Portuguese Republic [3], as well as by the Basic Health Law, approved by Decree-Law No. 095/2019 of 4 September. In order to guarantee this right and taking into account the recommendations set by the World Health Organization (WHO) [4], the Portuguese National Patient Safety Plan (PNSD) 2021–2026 [5] reinforces the need to endorse the importance of patient safety to consolidate and promote safety in healthcare provision. To this end, it emphasises the principles that sustains it, such as patient safety culture, communication, and the continued implementation of safe practices in environments that tend to be more complex [5].

Given the complexity and criticality of the healthcare provided, hospitals are institutions that are highly susceptible to incidents and adverse events [6,7]. In fact, recently, Chatzi and Malliarou [8] dove deeper into the definition of patient safety, claiming that it is the state in which harm to patients from nursing practice is eliminated or abridged so far as reasonably practicable through a continuing process of adverse events’ identification. The WHO refers to adverse events as errors or failures that occur during the provision of healthcare, which can result in harm to the patient, unrelated to their underlying condition [4,9]. These are unintentional and unexpected occurrences that can lead to temporary or permanent harm to patients and are one of the critical indicators of patient safety [10]. Studies estimate that 0.9% to 5.2% of deaths in hospital settings were potentially preventable [11]. In fact, the worldwide reporting of adverse events or incidents shows a very low prevalence of around 5% to 17%, of which 60% were preventable [12,13]. Evidence presented by the WHO [4] points to 134 million adverse events, contributing to 2.6 million deaths per year, so according to recent estimates, the social cost of patient harm is estimated at around USD 1 to 2 trillion per year. The Portuguese reality is in line with this trend, since 42% to 66% of the 11% of occurrences were preventable [12,13].

Patient safety culture is therefore a complex and multidimensional concept seen as the set of values, beliefs, norms, competencies, and individual and organisational behaviour patterns that determine commitment, style, and action regarding patient safety issues [2,4]. Referring to organisational behaviour, Fernandes and Queirós [14] highlight the enormous influence of nurses, since they are the largest professional group in Portuguese hospitals, and as a result of their job, have the most direct contact with patients. Given their proximity to patients, their recognised differentiated skills and their fundamental role in maintaining and consolidating a culture of patient safety [7,15], the professional nursing group is a key player in the process of continuously improving the quality of healthcare [15,16]. Thus, demonstrating nurses’ perceptions regarding patient safety culture can provide an overview of organisational conditions in this area and support for a more realistic analysis and understanding of individual and collective behaviour patterns that can have a positive or negative impact on healthcare. Therefore, this knowledge can enable the improvement of processes, flows, bundles, strengthening the focus on preventive measures, the notification of adverse events, and permanent and continuing education [17].

Several international studies point to various factors influencing nurses’ decisions to report adverse events, namely, the ability of the manager to respond, the fear of measures resulting from the report, the support of the team, and the existence or not of a safety culture [13,18,19,20]. Transposing this to the assessment of patient safety culture in national hospital institutions, the results are in line with the international overview [21]. In fact, the PNSD 2021–2026 is a reinforcement of the importance of promoting patient safety as part of a structured collective effort involving administrations, managers, and health professionals to raise public awareness of this issue [22]. In this regard, the Hospital Survey on Patient Safety Culture (HSOPSC), developed by the Agency for Healthcare Research and Quality (AHRQ) and translated and validated in Portugal by Eiras et al. [23], is a tool that allows a multidimensional assessment of safety culture [23,24]. In addition, the HSOPSC allows internal and external benchmarking exercises to be carried out: being widely applied in more than 93 countries, these provide a reliable international database on patient safety culture [24,25,26]. In Portugal, as a result of the evaluations carried out over the last 10 years, areas with potential for improvement have been identified, specifically the non-punitive response to error, the frequency of events reported, staffing, management support for patient safety, teamwork across units, communication openness, feedback and communication about errors, and overall perceptions of patient safety and transitions [26].

Although a transparent policy is currently advocated, with the recognition and analysis of reported adverse events, there is still a culture of blame in many organisations, discouraging professionals from reporting incidents, essentially due to concerns about liability and/or fear of their image in the eyes of their peers [27,28,29]. The consequences are under-reported and the expected learning from adverse events not occurring on a large scale [29]. Several studies found that supervisor/leadership responsiveness encompasses respect on the part of leaders and a sense of justice in the face of an employee’s exposure and reporting of an incident [30]. Similarly, a lack of feedback on the reported adverse event is seen as a factor in the reporting process [19] and the lack of time and nurses as an impediment to the adverse event reporting process [31].

This scarcity of adverse event records is a pattern in various health systems, resulting in biased knowledge of the facts and subsequently making it difficult to devise effective strategies [22]. The culture of reporting adverse events is a crucial element for critical patient safety culture (CPSC); therefore, involving healthcare professionals and their organisations in defining and implementing corrective and preventive interventions has a positive generative effect on incident reporting [31]. These and other initiatives contribute to strengthening the safety culture in healthcare organisations, constituting an important pillar for the sustainability of a learning environment and for building a sphere of trust in these organisations [32]. These authors also add that assessing CPSC allows continuous improvement processes to focus on the areas identified as priorities. In line with this, another study revealed that hospital units whose professionals rate patient safety more positively have an equally favourable assessment of healthcare satisfaction from the patients’ perspective [33]. These authors therefore state that investing in a patient safety culture can positively enhance the patient’s experience of healthcare.

This study arose from the need to promote a CPSC by analysing nurses’ perceptions on this matter, in order to draw up an overview of the organisational situation and define strategies for continuous quality improvement and risk management. That said, this study aimed to assess patient safety culture according to nurses’ perceptions in the context of providing healthcare to the people in critical condition (PCC) using the validated HSPSC, identifying associated factors and contributions to guiding its improvement. Based on the literature review, the following investigation questions were formulated. What is the perception of patient safety culture among nurses within the critical care environment? Are sociodemographic and professional characteristics associated with the CPSC?

## 2. Materials and Methods

### 2.1. Type of Study

This was an observational, cross-sectional study, written according to the Strengthening the Reporting of Observational Studies in Epidemiology (STROBE) recommendations [34] and using descriptive and inferential statistics.

### 2.2. Study Site

The hospital studied is a national health service (NHS) institution located in the centre of Portugal. The study focussed on the nurses at this institution whose departments included PCC, namely, the Intensive Care Unit (ICU), Operating Theatre (OR), and Emergency Department (ED).

### 2.3. Period

Data collection took place between September and December 2023.

### 2.4. Population

The population included the nursing teams of the ICU, OR, and ED, totalling 268 nurses, 11 of whom were absent due to illness or parenthood, giving a target population of 257 nurses.

### 2.5. Selection Criteria

The inclusion criteria were as follows: belonging to the nursing professional group, working in the units/departments being studied, and agreeing to take part in the study voluntarily.

### 2.6. Sample

The sample was non-probabilistic by convenience, as it included only nurses from the selected units/departments who volunteered to take part in the study.

### 2.7. Variables

The dependent variable corresponds to the CPSC dimensions and the independent variables to the sociodemographic and professional context variables (gender, educational qualifications, unit/department, experience in the unit/department, and experience in the organisation), as well as the variables of number of adverse event reports and perceived level of patient safety.

### 2.8. Data Collection Instrument

The questionnaire used corresponds to version 1.0 of the HSPSC, originally developed by the AHRQ [35], translated, validated, and provided by the authors [23] and adopted by the Direção-Geral da Saúde (DGS) under Regulation No. 005/2018 of 20 February 2018. The survey applied consists of two parts, the first of which covers the characterization of the sample, whose variables correspond to age, gender, educational qualifications, unit/department, years of experience in the unit/department, years of experience in the organisation, knowledge of whether the hospital/unit is accredited and/or is undergoing an external quality assessment process, and previous response to the survey.

The second part corresponds to the HSPSC, with 42 items, assessed using a Likert scale and evaluating 12 dimensions of patient safety culture: 1. teamwork; 2. supervisor/manager expectations and actions promoting patient safety; 3. organisational learning—continuous improvement; 4. management support for patient safety; 5. overall perceptions of patient safety; 6. feedback and communication about errors; 7. communication openness; 8. frequency of events reported; 9. teamwork across units/departments; 10. staffing; 11.transitions; and 12. non-punitive response to error [26,35,36]. In addition to these dimensions, the HSPSC also includes an assessment of the level of critical patient safety perceived by nurses and the number of adverse events reported in the last 12 months. It should be noted that all the questions were compulsory, and as such, no completed questionnaire was excluded for missing data.

### 2.9. Data Collection

The survey was made available electronically via institutional email, using Microsoft Forms^®^, sent through the Chief Nursing Officer of each unit/department within the scope of the study, along with a reminder to complete the questionnaire every month. Throughout the process, anonymity was guaranteed and only one response was given for each nurse, automatically linking the response submitted (which could not be filled in again) to the respondent’s institutional email address.

### 2.10. Data Processing and Analysis

To analyse and interpret the data, the indications in the AHRQ guide were used as a reference [35]. Given that the questionnaire includes negatively worded items (A5, A7, A8, A10, A12, A14, A16, A17, B3, B4, C6, F2, F3, F5, F6, F7, F9, and F11), the guidelines issued by the AHRQ suggest inverting the scale to facilitate analysis, and these items have been identified with an “r” (Table 1) [16,23,35,37].

Then, they were recoded and grouped into 3 categories in order to facilitate analysis: positive (encompasses the answers “agree”, “strongly agree”, “most of the time”, and “always”), neutral (includes the answers “neither agree nor disagree” and “sometimes”) and negative (“strongly disagree”, “disagree”, “never”, and “rarely”) [16,35].

Once the items had been grouped into the aforementioned safety culture dimensions [35], the “not applicable” answers (6 on the Likert scale) were omitted, the rating for each dimension was calculated to allow a proper comparative analysis between them and subsequently to analyse the possible statistical relationships between variables and trends through the respective median (Me). The AHRQ benchmark was also applied, which suggests that positive ratings of 75% or more indicate “strong” dimensions regarding safety culture, while positive responses of 50% or less correspond to weak dimensions that require improvements [17,38].

The data were analysed using SPSS^®^ version 29.0. In the field of inferential statistics, non-parametric tests are used, since the necessary postulates to apply parametric tests are not guaranteed and it is not possible to invoke the central limit theorem [39]. Therefore, the following tests were used: Kruskal–Wallis, Spearman, and Mann–Whitney U, considering a 95% confidence interval, as well as the use of frequencies (relative and absolute), standard deviation, mean, and median.

Concerning the analysis of the sociodemographic and professional characterization items, since the classes inherent to the variables “educational qualifications” and “unit/department” had frequencies of fewer than 5, they were grouped into other classes within their variable [39]. Thus, new variables emerge: the “professional category”, which includes the class of specialist nurse (combining the other speciality classes) and (generalist) nurse, and the variable “departments”, which includes 3 grouped classes (ED, OR, and UCI).

### 2.11. Ethical Aspects

Authorization was first sought from the authors who translated and validated version 1.0 of the HSOPSC for the Portuguese population. To carry out the study, it was essential to obtain a favourable opinion from the ethics committee, the legal support unit, and the board of directors of the institution under study.

Participation in this research required reading and accepting an informed consent form attached to the survey. In this way, participation was voluntary without prejudice to withdrawing at any time, guaranteeing the confidentiality of the data.

## 3. Results

### 3.1. Sociodemographic and Professional Characteristics

This study involved 57 (100%) nurses, 42 (73.7%) female, between 25 and 64 years old (38.74 ± 7.89). In terms of educational qualifications, the majority were general nurses (*n* = 44; 77.2%) and only 13 (22.8%) were specialists, including 5 (8.8%) with a specialization in medical–surgical nursing area in nursing to PCC. Average experience at the institution was 12.51 ± 9.04 years, with the chosen area being the Emergency Department (*n* = 35; 61.4%), where they had worked for an average of 12.02 ± 8.85 years. Regarding the accreditation process and/or external quality assessment process in the institution, 49 (86%) nurses said they were aware of it and only 2 (3.5%) of the sample had already answered the HSPSC survey. In the last 12 months, 33 (57.9%) of the respondents had not filled in any reports/notifications of incidents, 18 (31.6%) had filled in one to two reports and the rest had filled in three to five reports (*n* = 6; 10.5%) (Table 2).

Regarding the number of incidents/adverse events reported, there was a statistically significant correlation (*p* = 0.004) between this variable and the professional category, with specialist nurses being more likely to report (Table 3).

Concerning the item “level of patient safety”, 26 (45.6%) nurses assessed it in the context of their unit and answered “acceptable”, 20 (35.1%) nurses perceived the degree of patient safety as “very good”, and only 3 (5.3%) considered it “excellent”. Even so, a minority of five nurses (8.8%) considered the level of patient safety to be “poor” and three (5.3%) “very poor” (Table 4).

Considering a 95% confidence level, it was possible to ascertain a statistically significant relationship (*p* < 0.001) between the level of safety of critical patients in the organisation as perceived by the professional nursing group and the area in which they work, with the highest median corresponding to the ICU and the lowest to the ED (Table 5).

### 3.2. Dimensions of the CPSC

**Figure 1:**
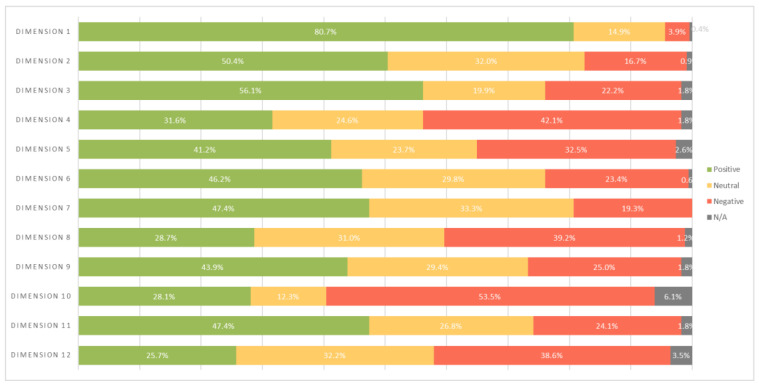
Average percentage of positive, neutral, and negative responses per dimension. N/A = not applicable; dimension 1. teamwork; dimension 2. supervisor/manager expectations and actions promoting patient safety; dimension 3. organisational learning—continuous improvement; dimension 4. management support for patient safety; dimension 5. overall perception of patient safety; dimension 6. feedback and communication about errors; dimension 7. communication openness; dimension 8. frequency of events reported; dimension 9. teamwork across units/departments; dimension 10. professional staffing; dimension 11. transitions; dimension 12. non-punitive response to error.

In terms of the items in the dimensions analysed, it was found that only dimension 1 (teamwork) was positive above the predefined criteria, making it a strong dimension with around 80.7% of positive responses. Dimensions 2 and 3 (supervisor/manager expectations and actions promoting patient safety and organisational learning—continuous improvement, respectively) were identified as neutral. The remaining dimensions had all items with an average percentage below 50%, and as such, represent an opportunity for improvement, as shown in Figure 1.

In this study, a statistically significant difference was found between dimension 9 (teamwork across units/departments) and age (*p* = 0.010) (Table 6), with the distribution of scores being proportional to the highest median age of the nurses.

Table 7 shows that the variable “departments” has a statistically significant relationship (*p* < 0.05) with dimensions 1 (teamwork), 2 (supervisor/manager expectations and actions promoting patient safety), 3 (organisational learning—continuous improvement), 5 (overall perception of patient safety), 9 (teamwork across units/departments), and 10 (staffing).

## 4. Discussion

In order to facilitate the discussion of the results, similarly to the approach adopted by Eiras et al. [40], some of the dimensions were aggregated as follows: 2, 3, and 12 included management/leadership, 6 and 7 corresponded to communication, and 9 and 11 corresponded to inter-area coordination. This strategy made it possible to work on eight dimensions: general perceptions of patient safety, teamwork, inter-area coordination, event notification, management/leadership, communication, continuous improvement, and professionals [40].

The data relating to the dimensions shown above in Figure 1 are corroborated by the national study published by the DGS [2] highlighting the first pillar of the PNSD 2021–2026, which corresponds to safety culture, whose strategic objectives include a commitment to leadership, transparency, communication, learning from mistakes, improving the quality of healthcare, and a culture of non-blaming and accountability [41]. As the DGS declares, the evaluation of safety culture is not an end in itself, but the beginning of a process of continuous improvement [2].

### 4.1. Overall Perception of Critical Patient Safety

With regard to the critical patient safety level, 45.6% of the sample considered this item to be “acceptable”, which is in line with what was obtained in dimension 5 (overall perception of patient safety), with around 41.2% positiveness and a median value (Me) of 3.00. This result indicates a clear need for improvement in this area, which has also been explained in other studies, including international ones [16,17,42,43,44,45,46,47,48,49]. However, there is a slight discrepancy with the results published by the DGS, reflecting a more positive perception of patient safety [2,41]. The positive perception of level of critical patient safety suggests that nurses are aware of the high-risk nature inherent in healthcare organisations that can lead to adverse events due to a sequence of systemic factors, including organisational strategies, work practices, culture, and risk prevention [50]. In alignment with these facts, the majority (35.1%) considered that hospital management has patient safety as a priority (item F8).

In line with these findings, specifically the statistically significant differences between nurses’ areas of work and the perceived level of critical patient safety in the organisation, other studies have also found close links between the work environment and CPSC [51,52]. Alongside this correlation, which to some extent falls within dimension 5 (overall perception of patient safety) it was found that dimensions 1 (teamwork), 2 (supervisor/manager expectations and actions promoting patient safety), 3 (organisational learning—continuous improvement), 9 (teamwork across units/departments), and 10 (staffing) were directly associated with the work areas/departments.

### 4.2. Teamwork

Considering the result obtained in relation to dimension 1 (teamwork), which is stated to be strong (80.7%; Me = 4.00), it is clear that there is an interpersonal and professional tendency to improve the care environment [14]. As Campelo et al. [53] state, good dynamics between elements, mutual support, and respect have a positive impact on nursing care. Corroborated by several national and international studies, this dimension can be considered an enabler of CPSC [2,16,17,42,54].

### 4.3. Inter-Area Coordination

Dimension 9, which refers to teamwork across units/departments, represents a weak area in need of improvement (43.9%; Me = 3.25), a result shared by the DGS and other international studies [2,7,41,42,55,56]. It should be noted that the influence of age on this dimension was not validated by Brás et al. [16], nor was the correlation between the age variable and the other dimensions verified in this study, differing from what was obtained by these authors. On the other hand, dimension 11, referring to shift transitions and hospital transfers, with around 47.4% (Me = 3.25) of positivity, shows an area of the CPSC in need of improvement. Brás et al. [16], similarly to these results, justify it by the fragmentation of health systems, which leads to an increase in transfers of patients (intra and inter-hospital) and thus a greater likelihood of errors in the information transmitted.

### 4.4. Notification of Events

As in other studies, more than half of the nurses surveyed did not report adverse events, as shown by dimension 8 (frequency of events reported) with low positivity (28.7%; Me = 3.00) [12,40,57,58,59]. This translates into little involvement by nurses in the reporting process, and as some authors point out, makes it difficult to devise effective preventive and corrective strategies [22,55]. This result is also shared by other national and international studies [40,60].

With regard to the number of events reported, it was found that specialist nurses were more likely to report them, as shown in Table 3. This is corroborated by the study conducted by Okuyama et al. [61], who found that the frequency of reported events differed significantly with educational qualifications, although was inversely proportional to education. Other authors also add that healthcare professionals with a master’s degree score higher in terms of patient safety culture than those with a bachelor’s degree [62]. In addition, Brás et al. [16] conclude that nurses with a bachelor’s/graduate degree, as well as nurses without a specialist title, show a more positive perception of patient safety culture in dimensions 3 (organisational learning—continuous improvement), 5 (overall perception of patient safety), 6 (feedback and communication about errors), and 8 (frequency of events reported), which was not substantiated in this study.

### 4.5. Management/Leadership

As fear of reprimand is one of the major factors identified as preponderant in the notification process [13,18,19,20], this feeling is indeed present among nurses, which can be perceived by the low positivity (25.7%; Me = 2.67) of dimension 12 (non-punitive response to error). This reality is also shared by other studies [42,44,54,57,63,64,65], suggesting a punitive culture is still present in healthcare organisations. These data are in line with the results obtained in the study carried out by the DGS, reflecting a low level of positivity (26%) in accordance with what was obtained in the above stated dimension [2]. Delving deeper into this dimension, a statistically significant relationship was found between age and item A16r (“Professionals wonder if their mistakes are recorded in their personnel file”(*p* = 0.048)), reinforcing the punitive culture, possibly associated with younger nurses’ concern about the negative image of their competence in the eyes of their peers and supervisors [28].

The expectations of the supervisor/manager and actions promoting safety to the PCC (dimension 2) demonstrate considerable positivity (50.4%; Me = 3.50), slightly above 50%, reflecting the active role of the manager in promoting CPSC. This requires a committed leader who cultivates motivation and a culture of learning by devising strategies and solidifying the foundations that support safe and effective processes [50]. Other studies are in line with these results, where support and understanding from the supervisor/leader are recognised by nurses [2,16,17,49,63,66,67,68]. Regarding management support for patient safety (dimension 4), with a positivity of only 31.6% (Me = 3.00), this reflects the perception that this commitment by the hospital management is below the nurses’ expectations, which is also supported by other studies [16,46,61]. Nevertheless, according to Order No. 1400-A/2015, it is essential to ensure that clinical boards, clinical and health councils, and quality and safety committees promote the adherence of their professionals to the evaluation of patient safety culture [21].

### 4.6. Communication

Feedback on the measures taken in response to a notification as well as other reasons inherent to underreporting, such as pressure from managers, work overload, forgetfulness, devaluing the error, and lack of knowledge on how to notify, are factors pointed out by Alves et al. [69], supporting the results obtained in dimension 8 (frequency of events reported), 6 (feedback and communication about the errors), and 7 (communication openness), with positivity in the order of 28.7% (Me = 3.00), 46.2% (Me = 3.33), and 47.4% (Me = 3.33), respectively. In fact, Hammoudi et al. [19] mention that the absence of feedback following a report is a major factor in the reporting process, since the participation of nurses who openly discuss strategies to mitigate errors and other adverse events shows a positive effect on the willingness to report incidents, corroborating the data from other studies [30]. Sharing this perspective, it is suggested that work processes be improved through effective communication, while others call for a more positive attitude on the part of peers, as it strengthens professionals’ willingness to report [19,70].

### 4.7. Continuous Improvement

Nurses assessed believe that in a learning culture, mistakes lead to positive changes within the healthcare organisation, as they gave dimension 3 (organisational learning—continuous improvement) a positive rating of approximately 50.4% (Me = 3.50). These data are congruent with other studies [48,53,55,71], emphasising that a culture of monitoring activities and feedback on safety results in reinforcing professional competence processes [16].

### 4.8. Professionals

Staffing (dimension 10) is also a critical element that can jeopardise the safety of critical patients, and was identified as weak (28.1%; Me = 2.50). In fact, in this dimension in particular, there was more marked negativity (53.5%), reflecting dissatisfaction with nurse:patient ratios, which is also shared by other international studies [44,48]. In line with this, Regulation No. 743/2019 of 25 September 2019 of the Portugal Order of Nurses [72] establishes that an adequate number of nurses, level of qualification, and skill profile are fundamental aspects for achieving safety and quality in healthcare.

### 4.9. Limitations of the Study

A limitation of this study is the fact that it is a non-probabilistic sample, which affects the representativeness of the data and the extrapolation of the results. Nevertheless, as seen in the study conducted by the DGS, the adherence rate was also considerably low, at around 22.2% (*n* = 57) [2]. This trend is corroborated by several studies, namely, by the DGS, which in 2020 obtained a national adherence rate of 13.8% [41], which may be indicative of the weak involvement of professionals and institutions in patient safety matters [2]. Another aspect, no less relevant, relates to the scarcity of studies on CPSC, making it difficult to discuss the results.

## 5. Conclusions

This study has made it possible to assess nurses’ perceptions of safety culture in units where patients are in critical condition. In general terms, the teamwork dimension stands out, having been identified as the only strong factor in the CPSC of the healthcare institution. Dimensions 2 (expectations of the supervisor/manager and actions promoting patient safety) and 3 (organisational learning—continuous improvement), although better positioned with positivity between 50% and 75%, should be the target of interventions so that they can be considered factors that enhance CPSC. On the other hand, the remaining dimensions assessed were below 50%, with the most emerging and requiring priority intervention being dimensions 12 (non-punitive response to error), 10 (staffing), 8 (frequency of events reported), and 4 (management support for patient safety).

The study carried out highlighted gaps related to the CPSC of the institution in question, helping to provide an overview in these matters. Weaknesses and positive aspects were identified, representing an opportunity for hospital management to adjust the risk management matrix and quality processes and flows so that strategies can be drawn up to promote and consolidate the CPSC.

The promotion and dissemination of the importance of a systematic evaluation of the CPSC in order to diagnose areas of improvement is fundamental in all healthcare organisations, guaranteeing the quality of healthcare to PCC. It is therefore recommended that a study be carried out regarding CPSC including a more representative sample, promoting adherence in advance through awareness-raising actions and strategies aimed at health professionals, and focusing on the dimensions identified as weak and therefore in need of improvement. In addition to what has been suggested, the promotion of a CPSC, in line with a process of continuous improvement through communication, training, and awareness-raising actions, must be evident and include the involvement and responsibility of top hospital management and leaders of healthcare institutions.

## Figures and Tables

**Table 1:** Dimensions of patient safety culture and corresponding items.

Dimensions	Items
1. Teamwork	A1, A3, A4, and A11
2. Supervisor/manager expectations and actions promoting patient safety	B1, B2, B3r, and B4r
3. Organisational learning—continuous improvement	A6, A9, and A13
4. Management support for patient safety	F1, F8, and F9r
5. Overall perception of patient safety	A10r, A15, A17r, and A18
6. Feedback and communication about errors	C1, C3, and C5
7. Communication openness	C2, C4, and C6r
8. Frequency of events reported	D1, D2, and D3
9. Teamwork across units/departments	F2r, F4, F6r, and F10
10. Staffing	A2, A5r, A7r, andA14r
11. Transitions	F3r, F5r, F7r, and F11r
12. Non-punitive response to error	A8r, A12r, and A16r

**Table 2:** Number of events reported by nurses in the last 12 months.

Number of Event Reports	*n*	%
None	33	57.9
1 to 2 event reports	18	31.6
3 to 5 event reports	6	10.5
6 to 10 event reports	0	0
11 to 20 event reports	0	0
21 or more event reports	0	0
Total	57	100

*n*—Number; %—Percentage.

**Table 3:** Association between number of events reported by nurses (*n* = 57) and professional category.

Variable	Number of Occurrences Reported		
	Mann–Whitney *U*	Wilcoxon *W*	*p*-Value
Professional category	152.500	1142.500	0.004

**Table 4:** Patient safety rating awarded by nurses.

Patient Safety Rating	*n*	%
Excellent	3	5.3
Very good	20	35.1
Acceptable	26	45.6
Poor	5	8.8
Very weak	3	5.3
Total	57	100

*n*—Number; %—Percentage.

**Table 5:** Association of level of patient safety attributed by nurses (*n* = 57) according to departments.

Variable	Number of Occurrences Reported	
	Kruskal–Wallis *H*	*p*-Value
Departments	16.968	<0.001

**Table 6:** Association between the sociodemographic variables (age, experience in the unit/departments and experience in the organisation) of nurses (*n* = 57) and the dimensions of patient safety culture.

Variable	Spearman Test		
	Age	Experience in the Unit/Department	Experience in the Organisation
	*p*-Value	*p*-Value	*p*-Value
Dimension 1	0.814	0.531	0.916
Dimension 2	0.753	0.250	0.514
Dimension 3	0.364	0.995	0.485
Dimension 4	0.085	0.634	0.726
Dimension 5	0.240	0.876	0.717
Dimension 6	0.433	0.421	0.421
Dimension 7	0.539	0.646	0.102
Dimension 8	0.347	0.598	0.832
Dimension 9	0.010 *	0.429	0.176
Dimension 10	0.258	0.725	0.606
Dimension 11	0.590	0.654	0.640
Dimension 12	0.190	0.230	0.376

* p < 0.05—Statistically significant.

**Table 7:** Association of the variable “hospital departments” (*n* = 57) with the dimensions of patient safety culture.

Variable	Departments (ED, OR and UCI)		
	Kruskal–Wallis *H*	df	*p*-Value
Dimension 1	16.035	2	<0.001 *
Dimension 2	9.360	2	0.009 *
Dimension 3	11.689	2	0.003 *
Dimension 4	3.925	2	0.141
Dimension 5	14.203	2	<0.001 *
Dimension 6	3.331	2	0.189
Dimension 7	1.132	2	0.568
Dimension 8	0.305	2	0.859
Dimension 9	7.687	2	0.021 *
Dimension 10	15.268	2	<0.001 *
Dimension 11	5.086	2	0.079
Dimension 12	0.684	2	0.710

* p < 0.05—Statistically significant; df—Degrees of freedom.

## Data Availability

Detailed data are available upon reasonable request to the corresponding author.

## Public Involvement Statement

No public involvement in any aspect of this research.

## Guidelines and Standards Statement

This manuscript was drafted against the Strengthening the Reporting of Observational studies in Epidemiology (STROBE) research.
